# “Now what?!” A practice tool for pharmacist-driven options counselling for unintended pregnancy

**DOI:** 10.1177/17151635211018716

**Published:** 2021-07-13

**Authors:** Nevena Rebić, Kristen Gilbert, Judith A. Soon

**Affiliations:** Faculty of Pharmaceutical Sciences, Vancouver, British Columbia; Faculty of Nursing, Vancouver, British Columbia; University of British Columbia, and Options for Sexual Health, Vancouver, British Columbia; Faculty of Pharmaceutical Sciences, Vancouver, British Columbia; Department of Family Practice, Vancouver, British Columbia

**Scenario:** If a patient suspects an unintended pregnancy, purchasing a pregnancy test from a local pharmacy is the most accessible method of addressing this concern. When the test comes back positive, the pharmacist may be the first person the patient consults for answers to the question: *Now what?!*

## The pharmacist’s role in supporting patients in sexual health initiatives and during pregnancy

- Each year in Canada, an estimated 40% of pregnancies are unintended, and the majority are related to imperfect contraceptive adherence.^1,2^ As frontline health care professionals, community pharmacists are often the primary point of access for patients to the health care system.^3,4^
- Observational data suggest that individuals with unintended pregnancies have less social support, higher levels of stress and more depressive symptoms compared to those with intended pregnancies.^5^
- Task-sharing and expanding the scope of practice of pharmacists, nurses and nurse practitioners are recommended to increase points of access to the health care system.^6^ Community pharmacists are optimally positioned to address this gap by conducting point-of-care pregnancy options counselling.
- A patient has 3 options after learning that they are pregnant: parenting, adoption or induced abortion.^7-9^ The role of the patient’s health care providers is to help them make an informed decision and connect them with resources providing medically accurate, timely and accessible information.^7-9^
- Pharmacists can connect patients experiencing an unintended pregnancy with community resources that provide nondirective pregnancy options care and referral to family planning and mental health services.^4^
- Internationally, pharmacists in a variety of practice settings have been increasingly taking on enhanced roles involved with sexual health–related counselling, often related to emergency contraception (EC), sexually transmitted infections (STIs) and HIV testing.^10^ Pharmacists are also becoming more engaged and confident in public, health-related counselling activities related to the promotion of healthy lifestyles, smoking cessation and sexual health services.^11^

## Professional responsibility

- The World Health Organization supports collaboration between health professionals involved in primary care in order to provide quality family planning services, eliminate unsafe abortion and promote sexual health.^12^
- Pharmacists counselling patients who have experienced an unintended pregnancy have a professional responsibility to provide care that is nondirective, without judgment and free from bias.^7,13,14^
- To ensure professional standards are met, it is important that pharmacists clarify their values regarding issues that may arise in this area, such as adolescent pregnancy, single parenthood and abortion, as well as identify personal beliefs that may interfere with their ability to support these patient demographics.^9,13,15^ For more specific information, pharmacists may refer to their provincial standards.
- If a pharmacist identifies that they cannot fulfil professional standards, they should have a referral process in place that does not create undue hardship for the patient or result in significant delays in the delivery of care.^7,13,14,16^
- A values clarification guide^17^ that assists health care providers in clarifying their personal beliefs about pregnancy options and examining how these intersect with their professional responsibilities can be found in Table 1.

**Table 1:** Resources for pharmacists

Singer J. Options Counseling: Techniques for Caring for Women with Unintended Pregnancies^15^ • Article that includes examples for “Starting points for discussion,” “Neutral responses” and a quick summary of the National Abortion Federation values clarification guide
Moss DA. Curbside Consultation: Counseling Patients with Unintended Pregnancy^16^ • Article includes a case scenario of a patient with an unintended pregnancy and provides guidance for how a primary care physician should approach this interaction and options counselling, including how to ask neutral questions and explore pregnancy options
The Abortion Option: A Values Clarification Guide for Health Care Professionals by the National Abortion Federation^17^ • Includes a historical summary of the law and regulations of abortion in Canada and the United States • Provides tools and reflection questions for clarifying your values about pregnancy and family planning as an individual and as a health care professional • Workbook available: https://prochoice.org/resources/the-abortion-option-a-values-clarification-guide-for-health-professionals/
Lowik A. Trans-Inclusive Abortion Services: A Manual for Operationalizing Trans-Inclusive Policies and Practices in an Abortion Setting^31^ • Includes recommendations on appropriate language use, creating a trans-inclusive health care setting and additional educational resources for patients and providers • Manual available: https://www.ajlowik.com/publications#/transinclusive-abortion
Trauma-Informed: The Trauma Toolkit^36^ • A toolkit to help service organizations and providers deliver trauma-informed care Includes recommendations for working with persons affected by different types of trauma (including sexual violence, the legacy of residential schools and the experience of immigrants and refugees) • Toolkit available: https://ccrweb.ca/en/trauma-informed-trauma-toolkit
Discussing Sexual Health, Substance Use and STBBIs: A Guide for Service Providers^57^ • Includes guides for how to discuss sexual and reproductive health topics with patients and additional educational resources on providing population-specific care • Available: https://www.cpha.ca/discussing-sexual-health-harm-reduction-and-stbbis-guide-service-providers

## Pregnancy options counselling

**Figure 1:**
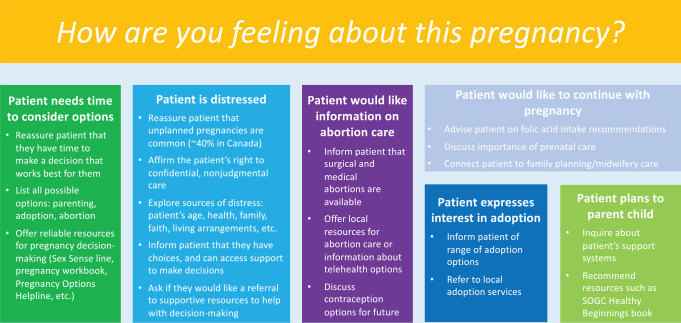
Options for Pharmacy Tool

- Pregnancy is a unique health care condition that permits multiple options that are clinically reasonable.^18^ Options counselling provides a patient with an unintended pregnancy the opportunity to explore their thoughts and feelings about the pregnancy.^13^
- It is essential that health care providers engage patients in nonjudgmental, nondirective and collaborative decision-making when discussing pregnancy options.^7,8,14^ A qualitative study examining what patients want their provider to discuss about pregnancy options reported that most patients supported options counselling to all pregnant patients, including discussions of parenting, adoption and abortion.^19^ Reasons mentioned included provider professional responsibility, respecting patient autonomy and avoiding assumptions about pregnancy intention.^19^
- Pharmacists are frequently approached by pregnant patients to provide advice about managing their medications during pregnancy and lactation.^20,21^ A recent intervention study among community pharmacists in Norway successfully provided consultations to pregnant women early in their pregnancy, with a special focus on the patient’s quality of life related to the management of nausea and vomiting.^22^
- Unintended pregnancies are associated with decreased likelihood of obtaining recommended amounts of preconception folic acid and first-trimester prenatal care as well as increased likelihood of postpartum depression and substance use pre- and postnatally.^7,18,19^
- Although specific guidelines for supporting pregnancy have not been outlined for pharmacists, best practices include conducting options counselling early in pregnancy, asking about the patient’s pregnancy intentions, articulating all options and providing accurate patient-oriented resources for optimal fetal care if the patient decides to continue the pregnancy.^9,23^
- It is essential for pharmacists to have an active role in this process, as inadequate involvement from a patient’s health care team can contribute to delayed care and subsequent increased risk of poor outcomes.^24^ For community pharmacists, this duty extends to the resources and referrals they provide to their patients.
- To facilitate this new counselling role for Canadian pharmacists, we have developed a novel Options for Pharmacy Tool (Figure 1).
- Resources for patients who wish to explore their pregnancy options are found in Table 2.

**Table 2:** Resources for patients

**Resources for pregnancy options information and provider referral**
Abortion Rights Coalition of Canada (ARCC): http://www.arcc-cdac.ca • Provides list of abortion clinics in Canada: http://www.arcc-cdac.ca/list-abortion-clinics-canada.pdf
Actions Canada for Sexual Health and Rights: https://www.actioncanadashr.org/ • Call national 24-hour access line: 1-888-642-2725 • Provides information on reproductive and sexual health and referrals on pregnancy options • Website provides list of service providers, including surgical and medical abortion, clinical and educational, and so on
National Abortion Federation (NAF): http://www.nafcanada.org/ • Call toll-free: 1-800-772-9100 (Monday-Friday 7 a.m. to 11 p.m.; Saturday-Sunday 9 a.m. to 9 p.m. Eastern Standard Time) • Provides answers to questions on abortion, unintended pregnancy or related issues, including financial assistance • For referrals to quality abortion providers in Canada: 1-877-257-0012
Sex and U by the Society of Obstetricians and Gynecologists of Canada: http://www.sexandu.ca/pregnancy/ • Offers broad overview of pregnancy options and links to resources • Full website has supplementary information on sexuality and sexual health
Sex-Sense—Options for Sexual Health: https://www.optionsforsexualhealth.org/sex-sense/ • Offers information and resources on sex, sexuality and sexual health provided by registered nurses, counsellors and sex educators • Within British Columbia and Yukon, call toll-free: 1-800-739-7367 (Monday-Friday 9 a.m. to 9 p.m. Pacific Time) • In Vancouver Lower Mainland, call: 604-731-7803 (Monday-Friday 9 a.m. to 9 p.m. Pacific Time)
The Pregnancy Options Workbook: http://www.pregnancyoptions.info • A workbook that provides a realistic picture of possible pregnancy choices, including abortion, adoption and being a parent, that a patient may independently review
**Resources for adoption**
Adoption Council of Canada: http://www.adoption.ca/ • Provides information on the adoption process and links to public and private adoption agencies by province
**Resources for pregnancy**
Our Sacred Journey: Aboriginal Pregnancy Passport by the Perinatal Services BC, Ministry of Health, and the First Nations Health Authority (FNHA) • Incorporates First Nations and Aboriginal traditional beliefs and values as well as clinical best practices • Provides health information, resources and traditional teachings • Passport available: https://www.fnha.ca/WellnessSite/WellnessDocuments/AboriginalPregnancyPassport.pdf
Pregnancy Info by the Society of Obstetricians and Gynaecologists of Canada: https://www.pregnancyinfo.ca/
**Emotional support talk lines and decision support**
All-Options: https://www.all-options.org/ • A confidential talk line for before and after abortion, adoption and parenting • Call toll-free: 1-888-493-0092 (Monday-Friday 10 a.m. to 1 a.m., Saturday-Sunday 10 a.m. to 6 p.m. Eastern Standard Time)
Exhale: www.4exhale.org • Call toll-free 1-866-439-4253 or text 617-749-2948 (check website for hours) • After-abortion text line and talk line that provides emotional support, resources and information

## Unique needs of patients with historically underserved and marginalized identities

- Pharmacists should be conscious of the unique needs of patients with historically underserved and marginalized identities, including adolescents; trans, lesbian, gay, bisexual, queer or questioning and Two-Spirit (LGBTQ2+) persons; Indigenous persons; persons with developmental or physical disabilities and persons who are survivors of violence.^7,13,25^
- Youth who have the capacity to provide consent do *not* need parental consent for medical treatment, including abortion, in Canada.^26^ Longitudinal research suggests that younger patients and adolescents do not demonstrate poor judgment when considering pregnancy options^27^ and should be involved in shared decision-making.^18^ Refer to the SOGC Adolescent Pregnancy Guidelines for more details.^25^
- The ability of an adolescent to consent to sexual activity should be considered and may necessitate reporting to child protection authorities if a sexual relationship violates the law.^25^ The general age of consent for sexual activity in Canada is 16, with ﻿age-related exceptions for adolescents between 12 and 16 years.^25^
- Transgender and gender-diverse individuals assigned female sex at birth may experience intended and unintended pregnancies after undergoing gender-affirming treatments, and many want to experience future pregnancy and parenthood.^28,29^ As these individuals routinely face discrimination navigating the health care system and may avoid seeking care due to fear of mistreatment,^30^ it is critical for health care professionals to incorporate practices that mitigate stigma and promote gender affirmation in patient care settings. Refer to the Trans-Inclusive Abortion Services resource guide for incorporating trans-inclusive policies and practices in your practice setting.^31^
- While Indigenous patients experience similar rates of unintended pregnancy as non-Indigenous patients,^32^ they face greater barriers to health and well-being as well as reduced access to health care services and culturally safe care, a recognized result of historical and ongoing forms of colonization.^33,34^ It is the responsibility of health care providers to ensure they are educated in providing culturally safe and competent care to Indigenous persons.^33^
- Persons with developmental or physical disabilities may require information on how their conditions may affect continuing a pregnancy in order to make an informed decision.^13^
- Contraception sabotage and pregnancy coercion may be common among individuals in violent relationships.^35^ Patients who disclose a history of violence or trauma warrant specialized care, including referrals for additional counselling.^13^ Refer to the Trauma Toolkit for strategies of delivering trauma-informed care.^36^
- Overall, options counselling should be culturally sensitive and accommodate for considerations of primary language, health literacy, gender identity, ethnicity and immigrant status.^13^

## Evidence-based strategy for pregnancy counselling

- Optimal evidence-based approaches for pregnancy options counselling in the community pharmacy setting have not been identified, but interdisciplinary and international resources can be used to inform pharmacy practice. A key strategy outlined by Simmonds and Likis^37^ is as follows:

1. Explore how the patient feels about the pregnancy and their options,
2. Help the patient identify support systems and assess risks,
3. Help the patient reach a decision or discuss a timetable for decision-making, and
4. Refer or provide the patient with appropriate services.

- Motivational interviewing can help patients reflect on feelings of ambivalence and take actions that align with their personal goals.^38^ Resources for approaching options counselling and providing sensitive reproductive care are found in Table 1.

## Abortion: Medical and surgical options

- In Canada, 31% of people who have the potential to get pregnant have had an abortion.^1^ In 2018, 85,195 abortions were reported in hospitals and clinics across Canada.^39^
- Abortion can be induced medically or surgically, with each option accompanied with unique risks and benefits.^2,7,8^ Both medical and surgical abortions have high success rates.^8^ Depending on the time since the patient’s last menstrual period, both medical or surgical abortion may be viable options, with the decision dependent on patient preference.^8^
- In July 2015, Health Canada approved the combination product mifepristone/misoprostol (Mifegymiso) for medical abortion up to 49 days since the last menstrual period and subsequently approved use to 63 days.^8,40^ There is evidence of effectiveness up to 70 days^8^ and in second-trimester abortion.^41^
- A Pharmacist Checklist and Resource Guide for Medical Abortion has been developed to provide a user-friendly practical tool to assist pharmacists with consistent counselling on the medications dispensed for medical abortion.^42^
- As primary care physicians can prescribe medications for medical abortion, medical abortion may be more accessible than surgery in rural and remote settings, as well as provide increased autonomy and privacy.^9^ Procedure bleeding and pain may be more pronounced than with surgery, and treatment may require more time to complete.^7,8^
- Telehealth options for medical abortion may be an appealing option during the COVID-19 pandemic and beyond, connecting patients remotely to an experienced provider for counselling, pregnancy diagnosis and follow-up care.^43,44^ The medications can be mailed to the patient or delivered to their pharmacy to be dispensed locally.
- Surgical abortion usually requires a clinic or hospital setting, which is frequently located in urban areas.^45^ The procedure often requires only a single visit and may be less painful, as anesthesia is offered.^7,8^ Patients have less autonomy due to procedural scheduling restraints and possibly decreased privacy due to needing to have help with transportation home following anesthesia.^7,8^
- Ovulation can occur as rapidly as 8 days following medical abortion.^46^ A contraception plan should be discussed to prevent subsequent unintended pregnancy.
- Following medical abortion, hormonal and progestin-only contraception can be initiated the day after misoprostol is taken.^8^ Long-acting reversible contraceptives (e.g., intrauterine device, implant) can be inserted at the follow-up visit with the prescriber.^8^
- Following surgical abortion, intrauterine devices can be inserted immediately afterward for ongoing contraception.^47^ Combined and progestin-only hormonal contraception can also be initiated immediately following surgery.^48^
- There is no abortion law in Canada,^17,49^ but the procedure is governed by provincial medical regulations, such as those related to gestational limits. All fees for surgical abortion are covered under provincial health plans for residents.^50,51^ Coverage for medical abortion depends on the provincial policy for mifepristone/misoprostol, with all Canadian provinces and territories, except Nunavut, providing universal coverage for residents as of June 2019.^52^ For individuals without provincial medical coverage, such as out-of-province patients or non-Canadian visitors, the out-of-pocket medication costs are between $300 and $450.^53^

## Adoption process

- Adoption is the formal transfer of care of a child, including relinquishment of all parental rights and responsibilities.^7,8^ In Canada, adoption services are mandated provincially, and legislation, policies and procedures vary in each province or territory.^54^ Adoptions can be facilitated by a public or private licensed agency and differ in the amount of contact information shared with adoptive parents.^54^
- It is important to recognize the historical trauma and ongoing impacts of Indigenous children being forcibly removed from their families in Canada, including the adoption of Indigenous children into non-Indigenous homes, leading to the separating of Indigenous people from their communities, culture and land.^55^ *Custom adoption*, a term that refers to the customary caretaking practices that have always been present in Indigenous communities, seeks to maintain Indigenous children’s connection to their diverse cultural, linguistic and spiritual identities.^25,55^
- With many adoption options available, including the level of “openness” related to parental involvement and information sharing, it is recommended that health care professionals understand the adoption process in their region to be able to guide their patients to appropriate resources.^18^ The Adoption Council of Canada provides regional information on the adoption process and is a good starting point: http://www.adoption.ca/.

## Antichoice agencies

- There are a number of antichoice agencies in Canada offering pregnancy counselling that use vague and misleading language to present their services as unbiased sources of medical information.^16,25,56^ These agencies, known as pregnancy crisis centres, are *not* licensed medical clinics.
- These agencies may provide false or misleading information and use religious pressure to persuade clients toward a specific option. Frequently, clients are not referred for contraception or abortion care.^56^
- As antichoice agencies may be the only resource available in rural areas, the pharmacist’s role as a nonjudgmental and informed counsellor is particularly important for delivering safe, medically accurate and culturally competent care.

## Conclusion

Pharmacists are increasingly becoming an essential part of facilitating access to reproductive health services and resources in their communities. As knowledgeable, accessible and approachable health care providers, pharmacists can use this practice tool to thoughtfully engage their patients in an informative and customized conversation about pregnancy options that support the specific needs of their patients. ■
